# Subcostal lymph nodes: An unusual sentinel lymph node basin in cutaneous melanoma

**DOI:** 10.1002/jso.27022

**Published:** 2022-07-23

**Authors:** Katherine M. Marsh, Courtney M. Lattimore, Christopher L. Cramer, Craig L. Slingluff, Lynn T. Dengel

**Affiliations:** ^1^ Department of Surgery University of Virginia Charlottesville Virginia USA

**Keywords:** melanoma, sentinel lymph node, subcostal lymph node

## Abstract

**Background and Objectives:**

Lymphatic drainage from subcostal nodes, along the costal groove, have not previously been characterized as sites for melanoma drainage and metastasis. This study reports a series of patients with subcostal nodes draining primary melanomas, with characterization of the sites of primary melanomas that drain to these nodes.

**Methods:**

Patients who presented to our institution between 2005 and 2020 with documented cutaneous melanoma and sentinel lymph node biopsy of a subcostal node (sentinel = S), or metastases to subcostal nodes later in clinical management (recurrent = R) were included. Patient demographics, melanoma pathology, nodal features, imaging information, surgical approaches, and outcomes data were collected.

**Results:**

Six patients had subcostal sentinel nodes (SNs). Primary sites included the posterior trunk and lateral chest wall. Subcostal nodes were found under ribs 10−12. Subcostal SNs had at least one dimension measuring 3 mm or less. There were no surgical complications related to removing the subcostal SN.

**Conclusions:**

Melanoma can metastasize to subcostal lymph nodes and be found at the time of SN biopsy or identified at recurrence. These small nodes are fed by lymphatic channels that run in the neurovascular bundle under the ribs. When lymphatic mapping identifies a subcostal SN, it should be excised.

## 1 INTRODUCTION

Sentinel lymph node biopsy (SLNB) is an essential procedure in the management and staging of malignant melanoma.^1^, ^2^ Preoperative lymphoscintigraphy is standard to determine lymphatic drainage and location of the sentinel lymph node(s).^3^ Cervical, axillary, and inguinal lymph nodes (LN) are the most common locations for the sentinel node (SN), but aberrant and atypical sites of lymph node drainage have been described. Less commonly, SN are located in the epitrochlear, popliteal, triangular intramuscular space, internal mammary, retroperitoneal, or mediastinal nodes, and among others.^4^, ^5^, ^6^, ^7^ In aggregate, these sites of unusual drainage account for 5%−22% of SNs.^5^, ^8^, ^9^ Those reports have helped to alert surgeons to the less typical locations, particularly the triangular intermuscular space nodes.^10^

Costal margin LN are another uncommon site of lymph node drainage, first identified in a series of 10 patients with periumbilical primary lesions. Two of these patients “had a lymph channel passing over the right costal margin.”^11^ These nodes were further described as subcutaneous SN overlying the costal margin, with lymphatic drainage continuing cephalad to the internal mammary nodes.^4^, ^11^, ^12^ In these reports of costal margin nodes, information was not provided about whether metastases were identified. To our knowledge and contrary to costal margin nodes, nodes under the rib in the costal groove have not previously been identified as potential sites of SNs nor further characterized in the literature. Management of these subcostal nodes, including the risk/benefit ratio of node resection versus the possible associated surgical complications, has not been defined.

The present report summarizes our institutional experience with identifying and managing subcostal sentinel LN in six patients. We present a single institutional experience series of LN found in the subcostal groove of lower ribs as SNs from the posterior trunk and flank. Importantly, metastatic disease was identified in 1/6 of these subcostal SNs. These data are presented including a discussion of appropriate patient management, and surgical considerations for safe removal of these nodes.

## 2 MATERIALS AND METHODS

A retrospective review of a prospectively collected database was performed and supplemented with information collected from patient medical records, in accord with IRB # 10803. All patients with cutaneous melanoma evaluated at our institution between 2005 and 2020 were eligible. Patients with documented SLNB of a subcostal SN or with a clinical recurrence in the subcostal node were included. Patient demographics, melanoma pathology, nodal features, imaging information, surgical approaches, and outcomes data were collected. Sentinel lymph node size was extracted from the operative report unless unavailable, in which case the measurements were taken from the surgical pathology record.

Patients were examined in two subgroups: (1) patients who had a subcostal node identified at the time of SN biopsy for their original lesion without any recurrence (sentinel = S) and (2) patients with melanoma recurrence in a subcostal node (recurrent = R).

To access subcostal SNs in the operating room, the latissimus muscle is split by separating the muscle vertically in the direction of its fibers. Then, the fascial attachments along the undersurface of the rib are divided. The subcostal neurovascular bundle can be identified in the groove on the inner inferior aspect of the rib, known as the costal groove or sulcus costae. One may have to reach under the bottom edge of the rib to access the node. Thus, operating in this area poses a risk to the associated intercostal nerve, vein, and artery. There is additional risk of either lung injury or pneumothorax. Thus, postoperative chest X‐rays were obtained in each patient who underwent subcostal node excision at our institution.

## 3 RESULTS

### 3.1 Patient population

Six patients with subcostal sentinel LN were identified and are referred to as S1−S6 (Table 1). Age ranged from 47 to 68 years. There were two females and four males; all were White.

**Table 1:** Clinical characteristics and measures for patients with subcostal sentinel nodes (SNs) identified at SN biopsy (S) or at recurrence (R)

Pt ID	Age/sex at dx	Month of diagnosis	Primary site	Histology breslow (mm) ulcer?	Month WLE/SLNB	Hot spots identified	SN^a^ total	Plus^a^ SN	Gamma counts of SN^b^	Subcostal SN dimensions (mm)	AJCC stage v8	Month last f/u	Status last f/u
**S1**	62 F	May 2006	Left lower back	SSM 1.4 No	June 2006	**Subcostal L 10th rib (2** a **)**	**1**	**0**	**1829**	**8 × 2 x 2**	cIB pIIIA	September 2015	Died
						Intramuscular near L 10th rib (1^a^)	1	0	1405	3 × 2 x 2			
						L flank deep SQ near L 10th rib (3^a^)	1	1	1299	4 × 4 x 4			
						Left axilla	1	0	1710	10 × 9 x 5			
						**Subcostal medial L 10th rib**	**N/a**	**N/a**	**351** a	**N/a**			
**S2**	68 M	October 2008	Left lower back	SSM 3.2 No	Dececmber 2008	**Subcostal L 12th rib**	**3**	**3**	**27 970**, **15 903**, **3566**	**6 × 3 x 2** **6 × 4 x 3** **2 × 2 x 1**	cIIA pIIIC	May 2013	Died with Dz
						Axillary	1	1	8962	9 × 5 x 6			
**S3**	62 F	September 2009	Left lower back/flank	SSM 2.2 No	November 2009	**Subcostal L 10th rib**	**1**	**0**	**4894**	**5 × 2 x 4**	cIIA pIIA	August 2018	Alive and well
						Axillary	2	0	35 962 4584	5 × 4 x 3 7 × 6 x 5			
						Intrathoracic	N/a	N/a	278^a^	N/a			
**S4**	67 M	January 2010	Left lower back	SSM 2.8 No	April 2010	**Subcostal L 11th rib**	**1**	**0**	**47 980**	**5 × 6 x 3**	cIIA pIIIA	May 2012	Alive and well
						L flank intransit (SQ)	1	0	13 092	9 × 7 x 6			
						L Groin	3	2	20 692 8713 4026	11 × 6 x 6 18 × 9 x 7 25 × 6 x 6			
**S5**	57 M	June 2020	Left inf lat lower back	Len 1.2 No	July 2020	**Subcostal L 12th rib**	**1**	**0**	**14 142**	**7 × 3 x 2**	cIB pIIA	July 2020	Alive and well
						Axillary	1	0	4936	10 ×4 x 3			
						Groin	1	0	10 093	15 × 10 x 8			
**S6**	47 M	September 2020	Right inf lat midback	SSM 1.3 Yes	November 2020	**Subcostal R 10th rib**	**1**	**0**	**36 329**	**8 × 7 x 3**	cIIA pIIIA	May 2020	Alive and well
						Axillary	1	0	4735	17 × 12 x 7			
						Axillary	1	1	8962	9 × 6 x 5			
**R1**	27 F	**March 2005**	Right lower back	N/a 3.5 No	March 2005	R groin	1	0	N/a	N/a	cIIA pIIA	November 2011	Died with Dz
						L groin	1	0	N/a	N/a			

*Note*: Node measurements done by surgeon in OR except pt S6 (surgical pathology gross measures). Bold indicates subcostal lymph nodes.

Abbreviations: Len, lentiginous; N/a, not available; SLNB, Sentinel lymph node biopsy; SLNV, sentinel lymph node biopsy; SSM, superficial spreading melanoma; WLE, wide local excision.

^a^ in situ count, node not excised.

^b^ max gamma counts of node ex vivo (per 10 s), except where noted

One patient was identified who recurred in a subcostal site. That patient (R1, Table 1) had primary melanoma diagnosed in March 2005 on the right lower back (Breslow 3.5 mm) and negative SN biopsies in bilateral groin at that time. That patient presented 3.5 years later with a mass in the right flank that the treating surgeon believed was a regional node in an atypical location, draining the primary site. The mass was located under the tip of the 12th rib and adjacent to it. It was resected en bloc with the tip of that rib. That patient was treated in the adjuvant setting on an experimental melanoma vaccine trial, developed recurrent disease in 2009 with additional lesions near the 12th rib resection and other distant disease. She was treated with high dose IL‐2 and with a BRAF inhibitor and died in November 2011.

### 3.2 Primary melanoma

All primary melanomas in patients S1‐S6 were located on the flank or back. The average Breslow depth was 2.2 mm (range 1.2‐3.2 mm). Two primary lesions were ulcerated (S1 and S6, Table 1). These patients underwent wide local excision of their primary melanomas with 1‐2 cm margins, per National Comprehensive Cancer Network (NCCN) guidelines.

### 3.3 Preoperative imaging

**Figure 1:**
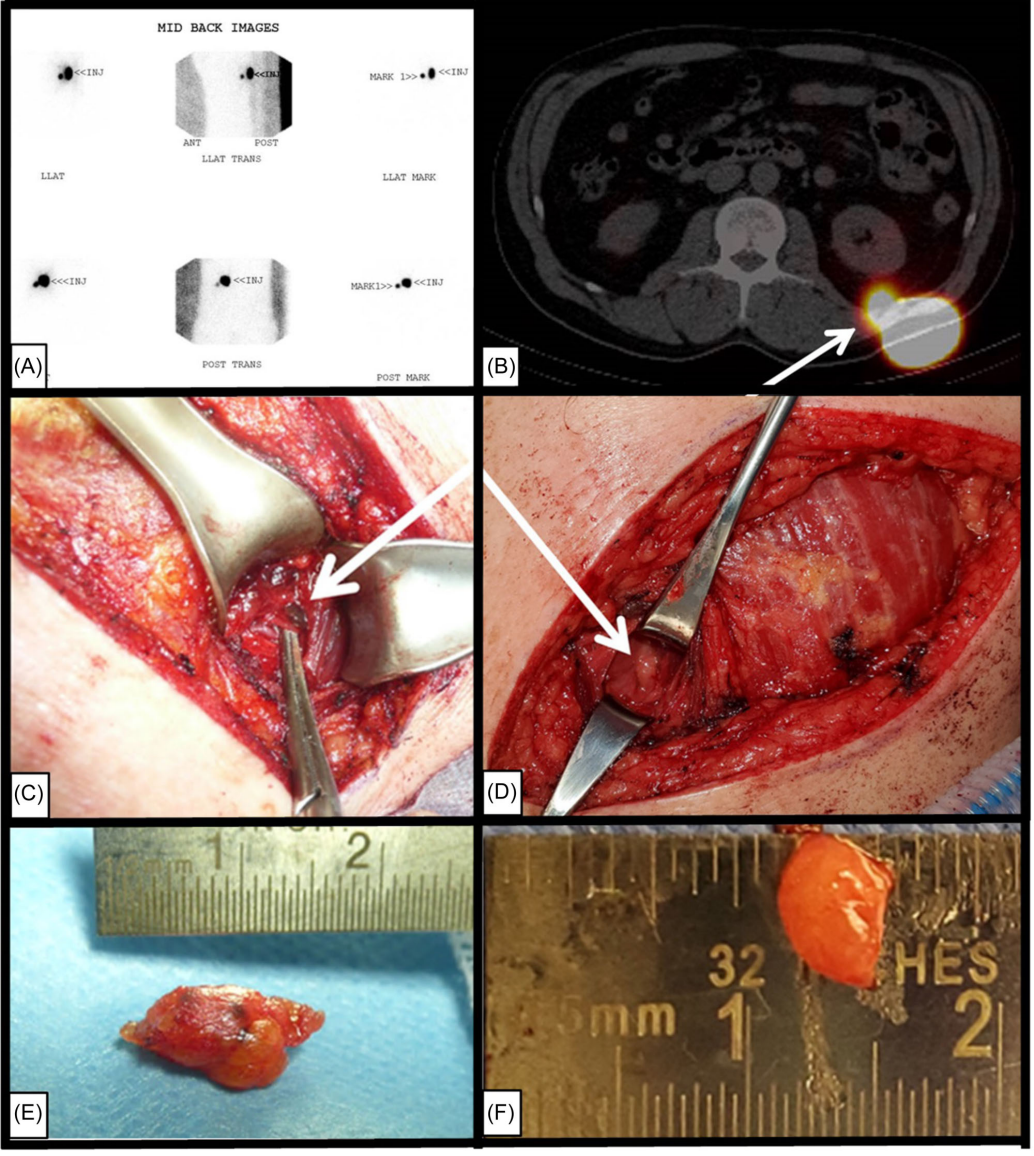
Pre, peri, and intraoperative imaging for patients S2 (A, C, E) and S5 (B, D, F). (A) lymphoscintigraphy showing a focus of activity noted lateral to the surgical site in the mid to lower back. (B) SPECT/CT revealing contiguous intense tracer activity immediately inferior to the left last rib. Intraoperative image of costal SN (arrow), inferior to the 12th rib (C, D), and intraoperative image of subcostal SN ex vivo (E, F). Melanoma metastasis was found in the subcostal SN shown in (C, E). SN, sentinel node; SPECT/CT, single‐photon emission computed tomography.

All patients in the SN group (Patients S1−S6) underwent preoperative lymphoscintigraphy at our institution using Technetium 99 m‐labeled sulfur radiocolloid injected intradermally at the site of the primary melanoma (example in Figure 1A). Blue dye was not used, in accord with our institutional practice.^13^ One or more subcostal SN was detected on each preoperative lymphoscintigraphy, allowing subcostal SN to be found before or during the index surgery.

One patient (patient S5) also underwent single‐photon emission computed tomography (SPECT/CT) at the time of initial lymphscintigraphy (Figure 1B). In this case, the subcostal SN was not initially visualized on the SPECT/CT due to the proximity of the node to the injection site. However, after the subcostal node was identified intraoperatively after wide excision of the melanoma and injection site. The radiologist was able to appreciate the node on retrospective rereview of the SPECT/CT (Figure 1B). Patient R1 was initially managed at another institution, and imaging findings were not available.

### 3.4 SN anatomy

**Figure 2:**
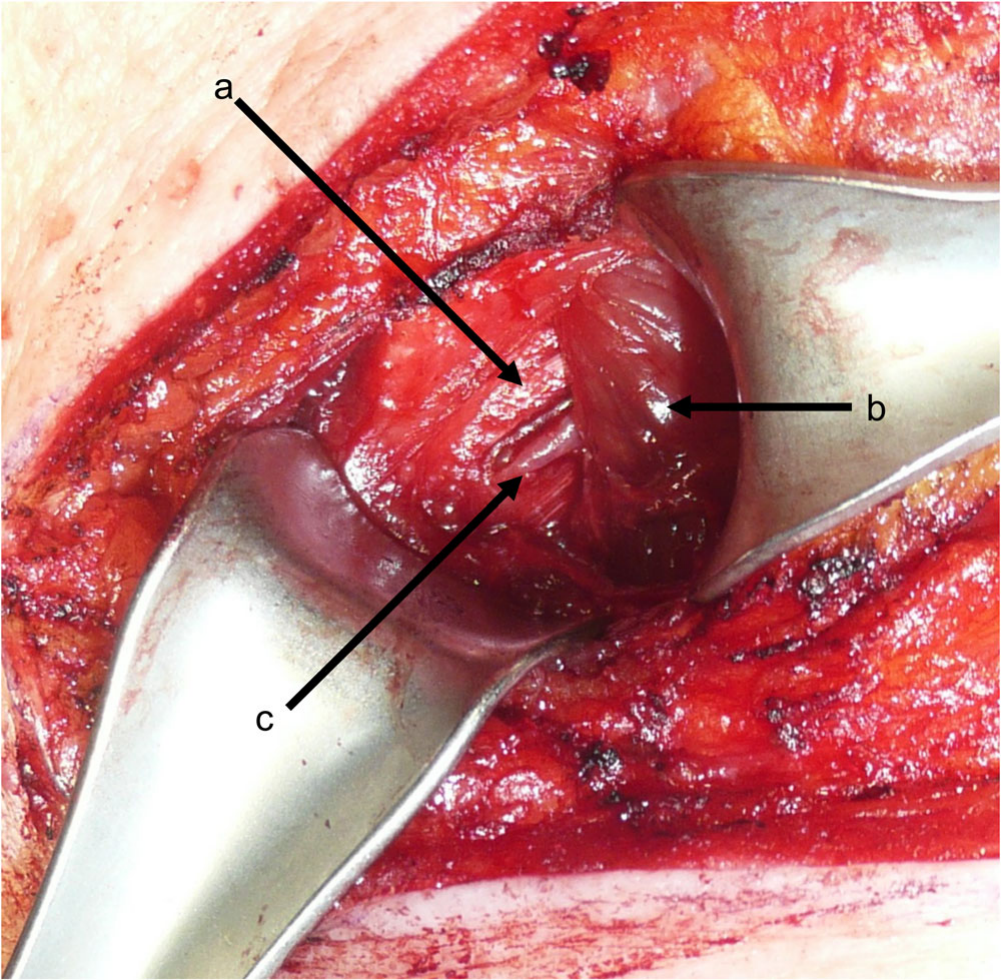
Surgical anatomy for a subcostal sentinel node (Patient S2). (a) inferior edge of left 12th rib, (b) fibers of latissimus dorsi (c) subcostal node adjacent to the subcostal vein in the 12th intercostal neurovascular bundle.

All subcostal SNs were located in the costal groove of the 10th to 12th intercostal ribs (Table 1), along the posterior or posterior‐lateral portion of those ribs. They were found anterior (more internal) and slightly superior to the bottom edge of the rib, along the lymphatic channels that run with the subcostal artery and vein (Figure 1B−D, Figure 2). Subcostal SNs measured 5–8 mm in maximal dimension and 2–3 mm in minimum dimension; all had at least one dimension that was 3 mm or less (Table 1, Figure 1E,F). Intraoperative count of the Tc^99^ sulfur colloid radiotracer uptake for subcostal SN ranged from 1829 to 47 980 (Table 1). One subcostal SN from Patient S2 appeared grossly pigmented on intraoperative inspection (Figure 1C,E) and contained metastatic melanoma on the pathology report.

In two cases in the SN group, presumed SNs were evident by imaging but were not removed because of difficulty exposing them safely. In both cases, they were deep to the paraspinal muscles (patient S1, S3; Table 1). Thus, the morbidity of possible thoracotomy required for node retrieval was thought to outweigh the benefit of resection. The SN pathology is therefore unknown. In both these cases, the nodes were surveilled with CT chest. Patient S1 remained clinically free of disease for 9 years, and then presented with diffuse bony metastases. Patient S3 continued to have negative surveillance CT scans (Table 1).

As noted above, for patient R1, the location of the presumed subcostal node metastasis was under the tip of the right 12th rib.

### 3.5 SN pathology

Five of six patients in the SN group had drainage to nonsubcostal lymph node basins in addition to the subcostal SNs. The other sites of SN included axillary (*n* = 4) and groin (*n* = 3). SN procedure included excision of the accessible subcostal SNs in all patients. Five of six patients in the SN group (S1, S3−6) and Patient R1 had drainage to nonsubcostal lymph node basins in addition to the subcostal SNs. The other sites of SN included axillary (*n* = 4: S1, S3−5), groin (*n* = 3: S4, S5, R1), and in an atypical site along the midaxillary line of the left flank (*n* = 1, S4, Table 1). One patient had 3/3 positive SN at the subcostal location (S2), in addition to a positive axillary node. Four patients with negative subcostal SNs had positive nodes elsewhere (Patients S1, S4, and S6).

### 3.6 Recurrence in the subcostal nodal basin

In this group of 7 patients, three (S1, S2, R1) developed recurrences during follow‐up. Patient S1 recurred 9 years following index operation with multiple osteophytic and osteoblastic lesions in the vertebral bodies, ribs, and sternum. Patient S2 had a positive axillary node along with 3/3 positive SNs in the subcostal region in 2008. He recurred near the subcostal site 1 year later, undergoing surgical resection of the adjacent ribs and soft tissue and radiation to the region. He eventually developed distant metastatic disease and died of disease 5 years after his initial diagnosis. Patient S2's recurrence was not thought to be due to tumor spillage but instead either possibly because of difficulty of excision in this complex anatomic location, or that more distant spread was already possible since he also had a positive axillary node. Patient R1 underwent routine surveillance PET/CT where recurrence at the right rib was detected, and as noted above, that patient also developed recurrences near the site of the rib resection, followed by distant metastatic disease.

## 4 DISCUSSION

This series demonstrates that small LN can be found along the neurovascular bundle in the costal groove under ribs 10−12, that lower back melanomas can drain to these nodes, and that metastases to these nodes can be found, either at the time of SN biopsy, or as a recurrence if they are not removed earlier. Thus, they should be sought when treating patients with lower back melanomas. These nodes may be identified with the aid of lymphoscintigraphy, SPECT‐CT scans, or possibly blue dye. Since the primary melanomas that drain to these sites may be near the subcostal regions, the injection site signal may obscure the hot spot at the subcostal SN. However, with a high level of suspicion they can be identified (Figure 1A,B).

The status of the SN remains to be the most important prognostic indicator in intermediate‐thickness melanoma,^14^ and SLNB remains a necessary cornerstone in staging and in determining optimal treatment.^1^, ^2^, ^15^ It may provide regional control similar to that with completion lymph node dissection.^2^ With increasing options for adjuvant therapies in node positive patients,^16^ it is important to obtain the most accurate assessment of SN status. Nodal drainage in melanoma is often not intuitive and includes atypical drainage patterns, or drainage to in‐transit nodes.^17^, ^18^ Improvements in preoperative lymphoscintigraphy and SPECT/CT imaging have enabled identification of SNs at unusual drainage sites.^19^ Previous literature has described “costal margin” SN in two patients as a drainage location solely from the anterior trunk or periumbilical regions,^11^, ^12^ suspected to be in‐transit nodes along the path to the internal mammary nodes.^4^, ^12^ Subcostal nodes are different, and as reported in this series, represent another atypical SN location, which differs from costal margin nodes by differences in the site of primary melanomas (lower back vs. anterior trunk and periumbilical skin), and without secondary drainage to internal mammary nodes.

Despite accurate detection with preoperative imaging, the small size of the subcostal SN can make their intraoperative localization challenging for the surgeon. The maximum subcostal node dimension in this case series was 8 mm (the smallest node measured only 5 mm by 2 mm by 4 mm) and all measured subcostal SN had at least one dimension measuring 3 mm or less. Awareness of the possibility and importance of these small subcostal SNs when reviewing preoperative imaging and during surgical resection is needed to assure SNs are not missed. Despite their small size, the intraoperative count of technetium 99 sulfur colloid radiotracer uptake was often similar to, or higher than, SNs in other basins (Table 1). The finding of recurrence in the subcostal location for patient R1, despite negative groin SN biopsies, suggests that metastasis to that location was likely present at the time of that SN biopsy but may have been missed due to low index of suspicion for a node in that site, or poor visualization of that node if the lymphatic channels may have been obstructed by tumor in the node. It is important to consider the operative risks of dissecting through the latissimus muscle to costal fascia and dissecting near the lung. Despite the potential risks, there was no reported morbidity including postoperative pneumothoraces or lung injuries in this series of patients with subcostal SNs. The morbidity of recurrence at this site, though, can be more significant as grossly present disease may require larger and more invasive resection.

The subcostal SN removed at the index operation for patient S2 was positive, and there was later recurrence near that site. Since he had a positive axillary node in addition to the three positive subcostal nodes, his higher disease burden on presentation could have contributed to the subcostal node recurrence. Patient R1 also had recurrences in soft tissue near the resected subcostal node metastasis. While her pathology results are unavailable since she was initially treated at another institution, it is possible her recurrence could have been a dermal or bony metastasis since the SLN drainage was to bilateral groins. These patients were treated before the systemic therapy options available today. Thus, for current patients presenting with positive subcostal SN at the time of SLNB, referral for systemic therapy should be pursued, in accord with standard practice. Furthermore, one patient underwent a partial chest wall resection because the subcostal node involved the adjacent rib (patient R1). With new awareness of the existence of subcostal SN, these patients with disease progression in the subcostal nodes may have been avoided with improved regional control by earlier removal of the subcostal SN at SLNB. Given the lack of complications and possible risks of expectant or delayed management, the fact that subcostal nodes can contain metastases (either at SLNB or at recurrence), and multiple patients presented with recurrent malignant melanoma at the subcostal site (Table 1), we recommend removal of these nodes at the time of SN biopsy.

## 5 CONCLUSIONS

For primary melanomas of the lower back, there should be a high suspicion for drainage to subcostal nodes so that they may be identified intraoperatively. Even if not evident on initial lymphoscintigraphy, they may be found intraoperatively with the gamma probe after the primary melanoma is widely excised. Subcostal SNs can be challenging to locate due to their small size and location abutting or beneath the associated rib. When lymphatic mapping identifies a subcostal SN, it should be excised along with other identified SNs, as the removal of subcostal nodes may alter staging, treatment and prognosis and possibly prevent late recurrence whose resection is more morbid.

## CONFLICT OF INTEREST

The authors declare no conflict of interest.

## SYNOPSIS

This study outlines a novel site for melanoma drainage and metastasis: subcostal lymph nodes. These small nodes are fed by lymphatic channels that run in the neurovascular bundle under the ribs, and should be excised when identified.

## Data Availability

The data that support the findings of this study are available from the corresponding author upon reasonable request.
